# Impact of Canal-Otolith Integration on Postural Control

**DOI:** 10.3389/fnint.2021.773008

**Published:** 2021-12-14

**Authors:** Andrew R. Wagner, Megan J. Kobel, Daniel M. Merfeld

**Affiliations:** ^1^Department of Otolaryngology—Head & Neck Surgery, Ohio State University Wexner Medical Center, Columbus, OH, United States; ^2^School of Health and Rehabilitation Sciences, Ohio State University, Columbus, OH, United States; ^3^Department of Speech and Hearing Science, Ohio State University, Columbus, OH, United States; ^4^Department of Biomedical Engineering, Ohio State University, Columbus, OH, United States

**Keywords:** vestibular, semicircular canal, otolith, noise, perception, sway, balance, postural control

## Abstract

Roll tilt vestibular perceptual thresholds, an assay of vestibular noise, have recently been shown to be associated with suboptimal balance performance in healthy older adults. However, despite the strength of this correlation, the use of a categorical (i.e., pass/fail) balance assessment limits insight into the impacts of vestibular noise on postural sway. As a result, an explanation for this correlation has yet to be determined. We hypothesized that the correlation between roll tilt vestibular thresholds and postural control reflects a shared influence of sensory noise. To address this hypothesis, we measured roll tilt perceptual thresholds at multiple frequencies (0.2 Hz, 0.5 Hz, 1 Hz) and compared each threshold to quantitative measures of quiet stance postural control in 33 healthy young adults (mean = 24.9 years, SD = 3.67). Our data showed a significant linear association between 0.5 Hz roll tilt thresholds and the root mean square distance (RMSD) of the center of pressure in the mediolateral (ML; *β* = 5.31, *p* = 0.002, 95% CI = 2.1–8.5) but not anteroposterior (AP; *β* = 5.13, *p* = 0.016, 95% CI = 1.03–9.23) direction (Bonferroni corrected α of 0.006). In contrast, vestibular thresholds measured at 0.2 Hz and 1 Hz did not show a significant correlation with ML or AP RMSD. In a multivariable regression model, controlling for both 0.2 Hz and 1 Hz thresholds, the significant effect of 0.5 Hz roll tilt thresholds persisted (*β* = 5.44, *p* = 0.029, CI = 0.60–10.28), suggesting that the effect cannot be explained by elements shared by vestibular thresholds measured at the three frequencies. These data suggest that vestibular noise is significantly associated with the temporospatial control of quiet stance in the mediolateral plane when visual and proprioceptive cues are degraded (i.e., eyes closed, standing on foam). Furthermore, the selective association of quiet-stance sway with 0.5 Hz roll tilt thresholds, but not thresholds measured at lower (0.2 Hz) or higher (1.0 Hz) frequencies, may reflect the influence of noise that results from the temporal integration of noisy canal and otolith cues.

## Introduction

Current models of postural control have implicated sensorimotor noise as one of the principal determinants of postural sway during quiet stance, with increases in sway attributed to increases in sensorimotor noise (Maurer and Peterka, 2005). While postural control has sensory and motor contributions, each with independent sources of noise, recent efforts in computational modeling suggest that postural sway is predominantly influenced by sensory noise, with limited contributions from noise in the motor pathways (van der Kooij and Peterka, 2011). Additionally, postural sway, even in conditions of impoverished visual cues, is under the influence of multiple sensory systems, including vestibular and proprioception. The influence of vestibular sensory noise on models of postural control has however been estimated to be approximately 10-times larger than the effect of noise in the proprioceptive system (van der Kooij and Peterka, 2011). Consistent with this notion, a recent empirical study of healthy older adults found that vestibular noise, assayed using vestibular roll tilt perceptual thresholds, was strongly correlated with the ability to complete a categorical (i.e., pass/fail) balance task (i.e., “eyes closed, standing on foam” Bermúdez Rey et al., 2016; Karmali et al., 2017); the mechanism underpinning the specific association between vestibular thresholds and reduced postural control has yet to be fully revealed.

Sensory noise denotes irregularities in neural activity which impairs one’s ability to perceive the accompanying afferent signal (Faisal et al., 2008). Vestibular afferent signals encode motion of the head in six degrees of freedom, with the semicircular canals encoding angular velocity (Fernandez and Goldberg, 1971) and otolith organs encoding gravitoinertial force (i.e., translation, and tilt; Fernandez and Goldberg, 1976). Due to imprecision in the transduction and subsequent transmission of the vestibular afferent signal (Faisal et al., 2008) the precision of self-motion estimates diminish as the signal to noise ratio decreases (Merfeld, 2011). Vestibular perceptual thresholds measure the size of a stimulus needed to exceed the baseline level of noise in the sensory system to enable reliable perception and thus have become a standard method for quantifying the level of vestibular sensory noise (Grabherr et al., 2008; MacNeilage et al., 2010; Merfeld, 2011; Valko et al., 2012; Agrawal et al., 2013; Bermúdez Rey et al., 2016; Crane, 2016; Kobel et al., 2021).

**Figure 1:**
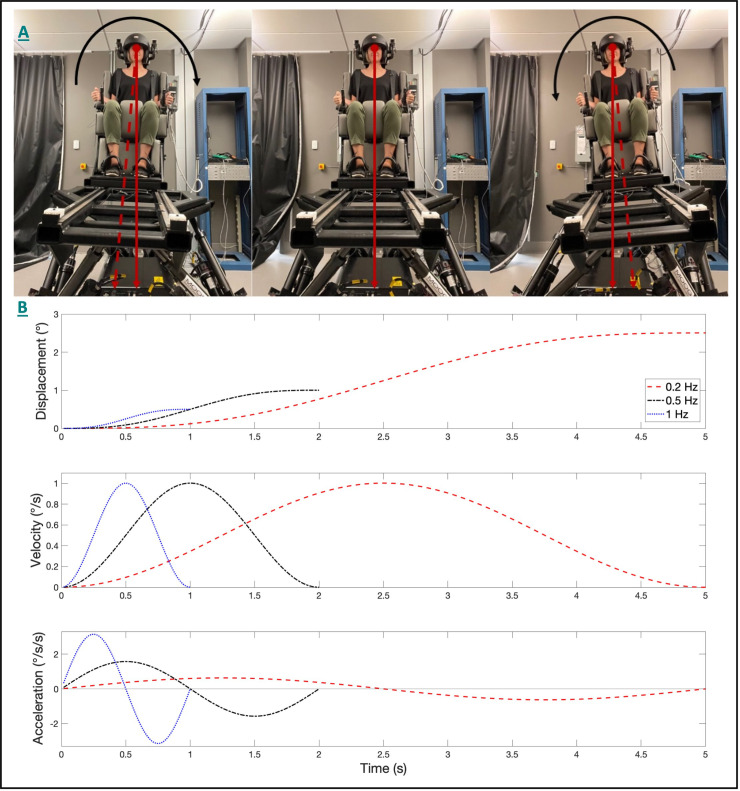
The 6DOF motion platform set up and the head-centered roll tilt motion is shown **(A)**. The angular displacement (top), velocity (middle), and acceleration (bottom) trajectories for single cycle acceleration stimuli are shown **(B)** for each of the three frequencies of roll tilt. The peak angular velocity was held constant at 1°/s for each condition. Note that for identical peak velocities, that displacements decreased, and peak accelerations increased as the frequency increased.

During dynamic roll tilt (Figure 1A), the canals and otoliths are each stimulated as the head rotates about an earth horizontal axis, with the otoliths encoding the resultant net gravitoinertial force. However, consistent with the behavior of all linear accelerometers (Einstein, 1907), on the basis of the afferent otolith signal alone, the brain cannot independently differentiate if the stimulus was due to a tilt (i.e., changing orientation relative to gravity) or translation (i.e., due to a linear acceleration inertial force) of the head (Angelaki et al., 1999). During roll tilt, angular velocity estimates derived from the vertical canals $\hat{\omega }$ must be temporally integrated $\left(\hat{G}=\int \left(-\hat{\omega }\times \hat{G}\right)dt\right)$ to yield a relative estimate of the orientation of gravity ($\hat{G}$) relative to the head (Glasauer, 1992; Merfeld et al., 1993, 1999; Angelaki et al., 1999; Merfeld and Zupan, 2002). Therefore, perceptual precision during roll tilt is reliant on the dynamic temporal integration of the canal signal with the otolith-derived estimate of gravity, with higher roll tilt vestibular thresholds indicating greater noise following this temporal integration. Accordingly, it has been proposed that the previously observed correlation between 0.2 Hz roll tilt thresholds and balance performance (Karmali et al., 2017; Beylergil et al., 2019) may represent the influence of noise resulting from the temporal integration of noisy canal and otolith signals on postural sway; however, this relationship has yet to be fully explored.

Earlier studies have compared categorical (i.e., pass/fail) balance assays, such as the “eyes closed, on foam” condition of the modified Romberg balance test (Agrawal et al., 2009), to roll tilt thresholds measured at 0.2 Hz and 1 Hz (Karmali et al., 2017, 2021). Due to unique dynamics associated with the processing of otolith (i.e., tilt; Fernandez and Goldberg, 1976) and canal (i.e., angular velocity; Fernandez and Goldberg, 1971) signals, their integration during such tasks is dependent upon the frequency of the roll tilt stimulus (Lim et al., 2017). In the absence of otolith cues, perceptual thresholds for earth vertical roll rotations (measured in supine) were found to plateau at frequencies above approximately 0.44 Hz (Lim et al., 2017); this behavior is qualitatively similar to the high pass filter characteristics of semicircular canal afferents (plateauing at approximately 0.03 Hz), with the higher cut off frequency for perception presumably reflecting an added influence of the central vestibular pathways mediating self-motion perception (Grabherr et al., 2008) or decision-making (Merfeld et al., 2016). Conversely, the perception of static tilt, as primarily mediated by the otoliths, is invariant with frequency (Lim et al., 2017), with sensitivity being proportional to the sine of the tilt angle (Fernandez and Goldberg, 1976; Jamali et al., 2019). As a result, for a fixed velocity, the otolith organs are stimulated to a greater extent at lower frequencies as the displacement is larger at lower frequencies of tilt (Figure 1B).

For dynamic roll tilt, Lim et al. (2017) used an optimal Kalman filter model to show that rotation cues and static tilt cues, presumably of canal and otolithic origin respectively, were optimally integrated at frequencies between 0.2 and 0.5 Hz, as measured thresholds were lower than predicted on the basis of a static combination of unimodal rotation and tilt cues (Lim et al., 2017). Accordingly, roll tilt thresholds within this range (i.e., 0.2–0.5 Hz) reflect contributions of noise associated with the canals, otoliths, and their dynamic temporal integration.

Here we measured roll tilt thresholds at the lowermost (0.2 Hz) and uppermost (0.5 Hz) ends of this range to assess the relative contributions of the canal and otolith cues. Due to the dependency of the otoliths on the amplitude of tilt, rather than frequency, their relative influence on roll tilt perception would be expected to be increased at 0.2 Hz, relative to 0.5 Hz, due to the increased displacement for a given velocity threshold (Figure 1). For 0.5 Hz roll tilt, the tilt amplitude decreases, and the higher frequency angular velocity cue leads to an increased reliability of vertical canal cues, due to the high pass nature of rotation perception. Thus, 0.2 Hz and 0.5 Hz roll tilt thresholds reflect noise resulting from canal otolith integration but differ in the relative precision of canal and otolith cues. We also assessed thresholds using a 1 Hz roll tilt stimulus to provide a measure of vestibular noise that resulted primarily from the vertical canals with minimal contributions from the otolith organs.

Our goal was therefore to determine if postural control is preferentially influenced by noise associated with the temporal integration of noisy canal and otolith cues (i.e., 0.2 and 0.5 Hz roll tilt). To address this question, we compared sensitive quantitative measures of quiet stance postural control to roll tilt vestibular thresholds measured at the frequencies previously used by others (0.2 Hz and 1 Hz; Bermúdez Rey et al., 2016; Karmali et al., 2021), as well as at 0.5 Hz, to determine if the frequency, and therefore relative influence of canal (0.5 Hz) and otolith (0.2 Hz) cues, influenced this relationship. We hypothesized that increased roll tilt noise, resulting from the temporal integration of noisy canal and otolithic cues, as represented by 0.2 and 0.5 Hz roll tilt perceptual thresholds, would be positively correlated with measures of postural instability measured in the corresponding mediolateral plane.

## Methods

### Participants

Since aging could impact balance *via* multiple age-related sensory and motor degradations, we tested only healthy young individuals so that we could quantify correlations between sway and tilt thresholds independent of the effects of aging. This substantially reduces the chance of a correlation between sway and tilt thresholds arising from any unmeasured age-related variation (e.g., age-related CNS declines) that might contribute to age-related changes in both tilt thresholds and sway. Data were collected on 33 healthy young adult volunteers (Mean 24.9 ± 3.67 years old, Range 20–32; 22/33 Female; Table 1). These individuals were recruited as part of a separate intervention trial, with a recruitment target of 30 participants. During this effort, two subjects dropped out during the intervention phase and were replaced, and due to time constraints, an additional subject agreed to only complete the baseline testing for the intervention trial; hence we report the baseline data here from 33 healthy participants. Each participant completed a health screening questionnaire prior to enrollment and denied any history of vestibular, neurologic, or alternative major medical comorbidity. The study was approved by the Ohio State University Institutional Review Board and each subject provided written informed consent prior to participation. All ethical standards set out in the Declaration of Helsinki were followed.

**Table 1:** Demographic information of participants and summary statistics for variables of interest.

N = 33 (22 female)	Mean	SD	95% CI	
Age (years)	24.9	3.67	23.60	26.21
Vestibular Thresholds (°/s)				
0.2 Hz	0.51	0.24	0.42	0.59
0.5 Hz	0.74	0.31	0.63	0.85
1 Hz	0.86	0.35	0.74	0.99
Vestibular Bias (°/s)				
0.2 Hz	0.018	0.12	−0.33	0.34
0.5 Hz	0.044	0.24	−0.55	0.58
1 Hz	0.02	0.21	−0.67	0.63
CoP—EC Foam				
ML RMSD (mm)	11.62	3.18	10.50	12.75
ML MV (mm/s)	33.23	8.93	30.06	36.39
ML MF (Hz)	0.36	0.12	0.32	0.40
AP RMSD (mm)	11.36	3.82	10.00	12.71
AP MV (mm/s)	32.64	9.24	29.37	35.92
AP MF (Hz)	0.35	0.14	0.30	0.40
CoP—EO Firm				
ML RMSD (mm)	4.25	1.38	3.76	4.74
ML MV (mm/s)	9.52	2.42	8.66	10.38
ML MF (Hz)	0.33	0.10	0.30	0.37
AP RMSD (mm)	4.54	1.72	3.93	5.15
AP MV (mm/s)	8.53	1.60	7.97	9.10
AP MF (Hz)	0.23	0.096	0.20	0.27

*Vestibular perceptual thresholds and biases for head-centered roll tilt motions representing the standard deviation and mean of the fitted cumulative distribution function, respectively. Mean CoP parameters for both the ML and AP directions are presented for each of two balance conditions of interest, “eyes closed, foam” and “eyes open, firm”. AP, anteroposterior; CoP, Center of Pressure; EC, eyes closed; EO, eyes open; ML, mediolateral; MF, mean frequency; MV, mean velocity; RMSD, root mean square distance*.

### Vestibular Thresholds

Vestibular self-motion perceptual thresholds were used to quantify vestibular perceptual noise (Merfeld, 2011). Subjects were positioned in a custom-made chair atop a 6DOF Moog (Elma, NY) motion platform (Figure 1A). A five-point seatbelt and a helmet were used to secure the subject to the chair and to mitigate unintended head movement while testing. Given the goal to quantify vestibular contributions to motion perception, all testing occurred in the dark to eliminate visual cues; directional auditory cues were masked with 60 dB SPL of white noise during each test motion.

Each of three test conditions consisted of 100 trials with the subject being tilted about a head-centered naso-occipital axis (Figure 1A) at a single discrete frequency (0.2, 0.5, or 1 Hz). The subject was instructed to indicate the perceived direction of the tilt stimulus (e.g., left or right) by pressing buttons held in the right and left hands (i.e., forced choice, direction recognition task). No feedback was provided, and subjects were instructed to make their best guess if unsure of the motion direction. Practice was provided until the subject reported feeling comfortable with the task. After each motion, a 3-s delay was provided prior to the next test motion to reduce the potential influence of motion after-effects (Crane, 2012). Due to the attentional demands of the task, subjects rested a minimum of 5 min between tests.

Consistent with past studies of vestibular perception (Grabherr et al., 2008; MacNeilage et al., 2010; Agrawal et al., 2013; Bermúdez Rey et al., 2016), we used single cycles of sinusoidal acceleration (Figure 1B) as the test stimulus. Dynamic roll tilts performed at 0.2 Hz, 0.5 Hz, and 1 Hz, therefore, correspond to motion stimuli having durations of 5, 2, and 1 s respectively. Single cycles of acceleration [(*a*(*t*) = *A*sin (2*πft*) = *A* sin (2*π t/T*); A = peak acceleration, *f* = frequency (i.e., the inverse of the duration (T) of the motion)] provide stimuli without discontinuities that mimic typical stimuli experienced during naturalistic human motion. The peak velocity (*v*_peak_ = *AT/π*) and peak displacement (*D* = *AT*^2^/2π) are proportional to the peak acceleration (A).

For the majority of trials, a standard four-down/1-up (4D/1U) adaptive staircase procedure was used in which the magnitude of the motion stimulus decreased each time the subject correctly reported the motion direction four times in a row (“4 down”), and the motion magnitude increased anytime the subject incorrectly reported the motion direction (“1 up”; Leek, 2001). A 2D/1U staircase was used until the first incorrect response to reach near-threshold stimulus levels more efficiently. Step sizes were selected using parameter estimation by sequential testing (PEST) rules (Leek, 2001). Using pilot data, we set the staircase to start at 5.5 degrees to ensure that each subject started at a suprathreshold stimulus.

Thresholds were calculated by fitting the binary subject responses (left/right) and the corresponding motion stimuli (direction and magnitude) to a Gaussian cumulative distribution function (CDF) defined by two parameters, the standard deviation (i.e., “threshold”) and the mean (i.e., “bias”). The threshold parameter represents the “one-sigma” vestibular threshold, as has been commonly reported (Valko et al., 2012; Bermúdez Rey et al., 2016; Karmali et al., 2021; Kobel et al., 2021), and represents: (1) the standard deviation of the underlying distribution function and (2) the stimulus level that would be expected to yield 84.1% accuracy in the absence of bias (Merfeld, 2011). Bias or “vestibular bias” (Merfeld, 2011) describes the displacement of the CDF along the abscissa; for example, a bias of +0.5°/s signifies that the individual would, on average, have an equal probability of reporting a right (negative) and a left (positive) rotation when the stimulus delivered is +0.5°/s (to the left; Merfeld, 2011). Estimates of threshold and bias were derived from a maximum likelihood estimate (Wichmann and Hill, 2001) using a bias-reduced generalized linear model (Chaudhuri and Merfeld, 2013) and probit link function. These methodological details have been published (Merfeld, 2011; Lim and Merfeld, 2012; Chaudhuri and Merfeld, 2013) and have been used extensively (Bermúdez Rey et al., 2016; King et al., 2019; Suri and Clark, 2020; Karmali et al., 2021; Kobel et al., 2021). We highlight that our bias reduced method accounts for the known serial dependency associated with staircase methods that have previously been shown to underestimate thresholds (Kaernbach, 2001; Klein, 2001; Chaudhuri and Merfeld, 2013). Given the attentional demands of the task, we also accounted for attentional lapses, defined as incorrect responses that occur independent of the magnitude of the motion stimulus, through the use of a lapse-identification algorithm using a standard delete-one jackknife when fitting the psychometric function (Clark and Merfeld, 2021).

### Postural Control

Center of pressure (CoP) data were collected from a tri-axial force plate (AMTI, Watertown, MA) at a sampling rate of 100 Hz. Subjects stood on a foam pad with the eyes closed and with feet in a narrow stance (i.e., medial border of the feet touching) for a duration of 63 s, with the first 3 s removed from the analysis to allow for the subject to accommodate to the conditions of the task; we used the same medium density (5 lb/ft^3^) foam pad (SunMate, 16”x18”x3”) that was used in the National Health and Nutrition Examination Survey (NHANES; Agrawal et al., 2009) and in the preliminary data relating roll tilt thresholds to “pass/fail” balance performance (Karmali et al., 2017). A secondary condition was captured where subjects were allowed to stand with their eyes open on a firm surface (while still in a narrow stance) for 33 s (with the final 30 s being analyzed). Alternative test conditions were performed as part of a larger data collection effort, however, our analysis focuses on these two tasks to provide: (1) a description of postural control when vestibular cues are known to dominate (eyes closed on foam), and (2) a control condition to determine if associations between vestibular noise and balance dissipate when vestibular cues are down-weighted in favor of visual and somatosensory cues (eyes open on a firm surface).

Maurer and Peterka (2005) found that CoP metrics aggregate into three independent groups—displacement, velocity, and frequency measures (Maurer and Peterka, 2005). To capture unique aspects of the postural control system, while also limiting the number of analyses, we *a priori* chose to focus our analyses on a single parameter from each of these three COP metric categories.

Root mean square distance (RMSD) is equivalent to the standard deviation of the zero-mean CoP tracing (Prieto et al., 1996); thus, it reflects the amount by which the CoP is displaced in a given plane of motion [anteroposterior (AP) or mediolateral (ML)], providing a quantitative metric of spatial control. Each measure was calculated separately in the ML and AP planes. In Equation 1, n is the total number of samples (60 s × 100 Hz = 6,000) and *x* represents the CoP displacement after removal of the mean (Equation 1).


$$x_{CoP}=CoP_{disp}-\left(\frac{1}{n}\sum_{i\;=\;1}^{n}CoP_{disp}\right);$$

(1) $$RMSD=\sqrt{\frac{1}{n}\sum_{i\;=\;1}^{n}{\left[x_{CoP}(i)\right]}^{2}}$$

Mean velocity (MV) describes the average instantaneous velocity of the CoP and is calculated by differentiating the CoP displacement signal (Equation 2).


(2) $$MV=\frac{1}{n-1}\sum_{i\;=\;1}^{n\;-\;1}\left[{\dot{x}}_{CoP}(i)\right]$$

Mean frequency (MF) uses the CoP velocity and displacement data to describe the oscillatory behavior of the CoP reflected as the number of cycles of CoP displacement per second (Hz; Equation 4). MD represents the mean distance of the CoP from the zero-meaned center of the CoP trajectory (Equation 3).


(3) $$MD=\frac{1}{n}\sum_{i\;=\;1}^{n}\left|x_{CoP}(i)\right|$$

(4) $$MF=\frac{MV}{\left(2\pi \;MD\right)}$$

As a secondary analysis, we set out to examine the relationship between the frequency content of the postural sway and the frequency of the roll tilt stimulus. We computed the one-sided power spectral density (PSD) of the mediolateral CoP data using Welch’s method (*pwelch*; MATLAB R2020b). The CoP tracing was divided into eight segments with adjacent segments overlapping by 50%; each segment was then windowed using a Hanning window. To avoid the influence of measurement noise, a frequency range of 0.01–20 Hz was used. The area under the PSD curve was calculated and the frequency at which 95% of the power fell below was determined. In addition to the individual PSD’s, a median PSD was also found by taking the median power at each discrete frequency.

### Data Analysis

For our primary analyses, each of the three principal CoP metrics (RMSD, MV, and MF) from the “eyes closed, on foam” condition was regressed on each of the three vestibular threshold measures (0.2, 0.5, 1 Hz), yielding nine univariate regression models. A Bonferroni correction was used to account for multiple comparisons (α = 0.05/9 = 0.006). In order to determine the effect of individual thresholds while controlling for shared elements of perceptual noise, we then constructed multivariable regression models whereby each of the three CoP metrics was regressed on all three threshold measures, in addition to age, yielding three regression models. The primary analyses focused on CoP metrics quantified only in the mediolateral plane given the shared direction with the roll tilt stimulus. However, to determine the directional specificity of the relationship between roll-tilt thresholds and mediolateral sway, the above analyses were repeated for CoP data in the anteroposterior plane.

Secondary regression analyses were completed to assess the relationship between the significant predictors of sway in the “eyes closed, on foam” condition and balance performance in an “eyes open, firm standing” condition, where vestibular contributions are minimal (Fitzpatrick and McCloskey, 1994). This was done to further test our central hypothesis that noise resulting from canal-otolith integration influences postural control in conditions where vestibular cues are prioritized (eyes closed, on foam) rather than in conditions where alternative sensory systems are known to dominate (eyes open, on firm).

Several studies have log transformed vestibular perceptual thresholds to achieve normality (Benson et al., 1989; Grabherr et al., 2008) prior to analysis. However, quantile-quantile normal probability plots and the Shapiro-Wilk test of normality showed that the residuals from each of our regression models failed to deviate significantly from a normal Gaussian distribution, so we did not transform our data. All analyses were completed using Stata (v 16.1, College Station, TX).

## Results

**Figure 2:**
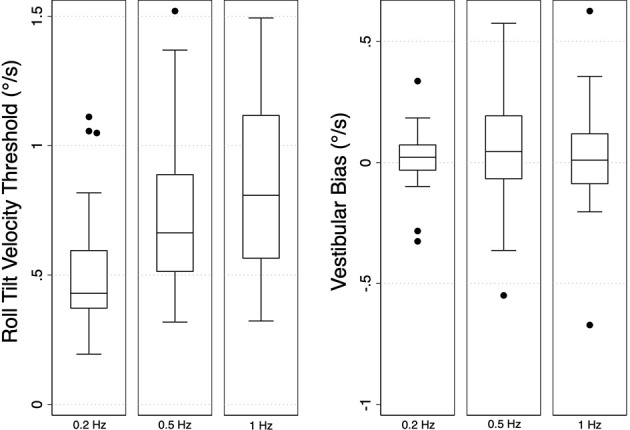
Box plots showing the median values and distributions of vestibular thresholds (left) and biases (right) for each frequency of head-centered roll tilt (*N* = 33). Velocity thresholds were found to increase with increasing frequency, while bias was similar at each frequency. Error bars represent ±1.5 times the first (Q1; 25th percentile) and third (Q3; 75th percentile) quartiles [interquartile range (IQR) = Q3 − Q1]. Outliers, defined as points greater than 1.5x the IQR, are shown as filled black circles.

Mean vestibular perceptual velocity thresholds and velocity biases, as well as our CoP parameters of interest for each condition (eyes closed, foam and eyes open, firm), are listed in Table 1. Overall, roll-tilt vestibular perceptual velocity thresholds increased with increasing frequency (Figure 2) consistent with past reports (Valko et al., 2012; Bermúdez Rey et al., 2016; Lim et al., 2017). At all frequencies, confidence intervals for measured biases included zero, showing no evidence of directional asymmetry.

**Figure 3:**
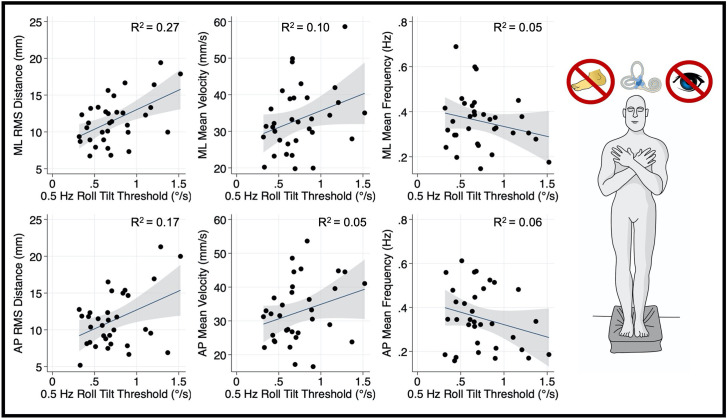
Relationship between 0.5 Hz roll tilt thresholds and RMSD, mean velocity, and mean frequency for both the mediolateral plane (top) and anteroposterior plane (bottom) during an “eyes closed, on foam” balance task. To the right of the plots, the task and relevant balance control systems are displayed, showing a dominance of vestibular cues in the “eyes closed, foam” condition. Each data point represents one individual’s performance. The blue line shows a linear fit with the 95% CI depicted by the gray shaded region. A statistically significant relationship between RMS distance and 0.5 Hz roll tilt was seen (upper left); not one of the five other associations plotted here was statistically significant. RMSD, root mean square distance.

In the “eyes closed, standing on foam” balance task, univariate linear regression models showed a significant linear association between mediolateral RMSD of the CoP and 0.5 Hz roll tilt thresholds (*β* = 5.31, *p* = 0.002, 95% CI = 2.1–8.5; Figure 3). While a positive association can be observed between 0.5 Hz roll tilt thresholds and the mediolateral MV, this effect failed to reach statistical significance (*β* = 9.13, *p* = 0.072, CI = −0.87–19.1). No significant relationship was seen between 0.5 Hz thresholds and the mediolateral MF (*β* = −0.09, *p* = 0.2, 95% CI: −0.22–0.05).

**Figure 4:**
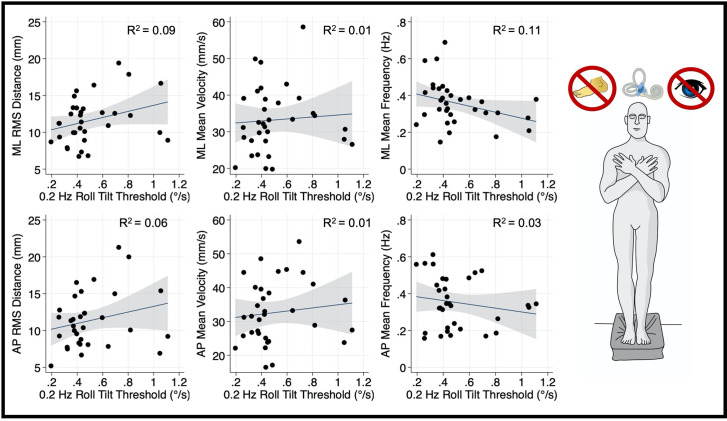
Relationship between 0.2 Hz roll tilt thresholds and RMSD, mean velocity, and mean frequency for both the mediolateral plane (top) and anteroposterior plane (bottom) during an “eyes closed, foam” balance task. To the right of the plots, the task and relevant balance control systems are displayed, showing a dominance of vestibular cues in the “eyes closed, foam” condition. Each data point represents one individual’s performance. The blue line shows a linear fit with the 95% CI depicted by the gray shaded region. No statistically significant relationships were seen between 0.2 Hz thresholds and postural sway. RMSD, root mean square distance.

**Figure 5:**
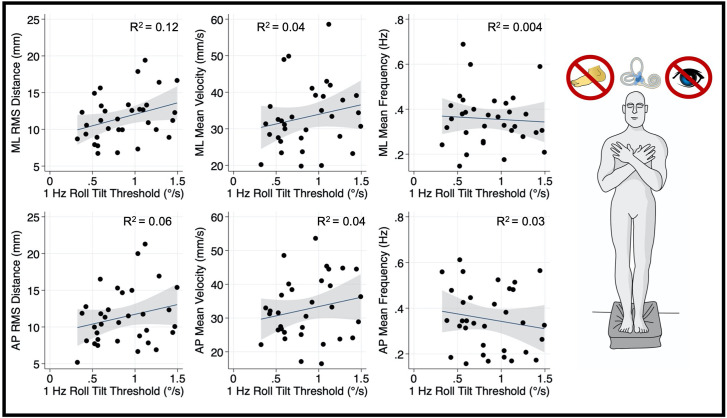
Relationship between 1.0 Hz roll tilt thresholds and RMSD, mean velocity, and mean frequency for both the mediolateral plane (top) and anteroposterior plane (bottom) during an “eyes closed, foam” balance task. To the right of the plots, the task and relevant balance control systems are displayed, showing a dominance of vestibular cues in the “eyes closed, foam” condition. Each data point represents one individual’s performance. The blue line shows a linear fit with the 95% CI depicted by the gray shaded region. No statistically significant relationships were seen between 1.0 Hz thresholds and postural sway. RMSD, root mean square distance.

Thresholds measured at 0.2 Hz (Figure 4) and 1 Hz (Figure 5), where otolith and canal cues respectively are more reliable, were not significantly associated with mediolateral MV (0.2 Hz: *β* =2.7, *p* = 0.69, CI −11.1–16.6; 1 Hz: = 5.26, *p* = 0.256, CI −4–14.5), or mediolateral MF (0.2 Hz: *β* = −0.16, *p* = 0.065, CI −0.34–0.01; 1 Hz: *β* = −0.0222, *p* = 0.72, CI −0.15–0.1). A positive trend was seen between both 0.2 Hz and 1 Hz thresholds and mediolateral RMSD, however, these associations did not reach statistical significance (0.2 Hz: *β* = 4.13 *p* = 0.083, CI −0.57–8.83; 1 Hz: *β* = 3.12, *p* = 0.053, CI: −0.045–6.3; Figures 4, 5, respectively).

After correcting for multiple comparisons, postural sway in the anterior-posterior plane orthogonal to the plane of motion for roll tilt thresholds was not associated with roll tilt perceptual thresholds at any frequency (α > 0.006; Figures 3–5). However, there was a positive linear trend between 0.5 Hz roll tilt thresholds and anterior-posterior RMSD of the CoP (*β* = 5.13, *p* = 0.016, CI: 1.03–9.23) that was not statistically significant.

To ascertain if the relationship between postural control and 0.5 Hz thresholds was driven by elements shared between the three threshold measures (including individual elements of canal and otolith noise) multivariable regression analyses were completed. The significant positive relationship between 0.5 Hz thresholds and mediolateral RMSD persisted (*β* = 5.44, *p* = 0.029, CI = 0.60–10.28), while no significant relationship was seen for 0.2 Hz or 1 Hz thresholds (Table 2); this finding may reflect an association between postural control and the noise inherent to the temporally integrated canal-otolith signal. Similar to the univariate analyses, a positive trend between 0.5 Hz thresholds and mediolateral MV was observed but did not reach statistical significance (Table 3). Neither 0.2 Hz nor 1 Hz roll tilt thresholds showed a significant effect on any of the postural control measures (Tables s 2–4) except a statistically significant relationship between the mediolateral MF and both 0.2 Hz thresholds and age was identified (Table 4). However, as both effects were small, and 0.2 Hz thresholds were negatively associated with mediolateral MF, the importance is unclear. In addition, larger samples have shown that thresholds are stable under age 40, and thus, the weak, positive effect of age may result from sampling variability. When compared to CoP data measured in the AP direction, similar to the univariate analyses, no significant effects were observed between any of the threshold measures when regressed on each of the CoP metrics. While not significant, there was a positive relationship between 0.5 Hz roll tilt thresholds and AP RMSD (*β* = 5.91, *p* = 0.057, 95% CI = −0.2–12.03).

**Table 2:** Results of a multivariable linear regression model.

ML RMSD	β	SE	t	p	95% Conf. Interval		Sig.
0.2 Hz Roll Tilt	−0.48	2.791	−0.17	0.865	−6.197	5.238	
0.5 Hz Roll Tilt	5.442	2.361	2.30	0.029	0.605	10.278	*
1 Hz Roll Tilt	0.065	1.941	0.03	0.974	−3.91	4.04	
Age	0.107	0.145	0.74	0.464	−0.189	0.404	
Intercept	5.122	3.72	1.38	0.179	−2.498	12.742	

Controlling for 0.2 Hz and 1 Hz roll tilt thresholds, as well as age, 0.5 Hz roll tilt thresholds displayed a significant positive effect on the mediolateral RMSD in the “eyes closed, foam” condition. RMSD, root mean square distance; ML, mediolateral. *Significant at alpha < 0.05.

**Table 3:** Results of a multivariable linear regression model.

ML MV	β	SE	t	p	95% Conf. Interval		Sig.
0.2 Hz Roll Tilt	−9.747	8.408	−1.16	0.256	−26.969	7.476	
0.5 Hz Roll Tilt	12.874	7.112	1.81	0.081	−1.695	27.443	
1 Hz Roll Tilt	0.483	5.845	0.08	0.935	−11.491	12.457	
Age	0.598	0.436	1.37	0.181	−0.295	1.491	
Intercept	13.348	11.205	1.19	0.244	−9.605	36.301	

*None of the roll tilt threshold measures were found to have a significant effect on the mediolateral MV in the “eyes closed, foam” condition. MV, mean velocity; ML, mediolateral*.

**Table 4:** Results of a multivariable linear regression model.

ML MF	β	SE	t	p	95% Conf. Interval		Sig.
0.2 Hz Roll Tilt	−0.223	0.106	−2.10	0.045	−0.441	−0.006	*
0.5 Hz Roll Tilt	−0.035	0.09	−0.39	0.697	−0.219	0.149	
1 Hz Roll Tilt	0.059	0.074	0.80	0.43	−0.092	0.211	
Age	0.012	0.006	2.17	0.039	0.001	0.023	*
Intercept	0.148	0.142	1.04	0.306	−0.142	0.438	

None of the roll tilt threshold measures were found to have a significant effect on the mediolateral MF in the “eyes closed, foam” condition. MF, mean frequency; ML, mediolateral. *Significant at alpha < 0.05.

**Figure 6:**
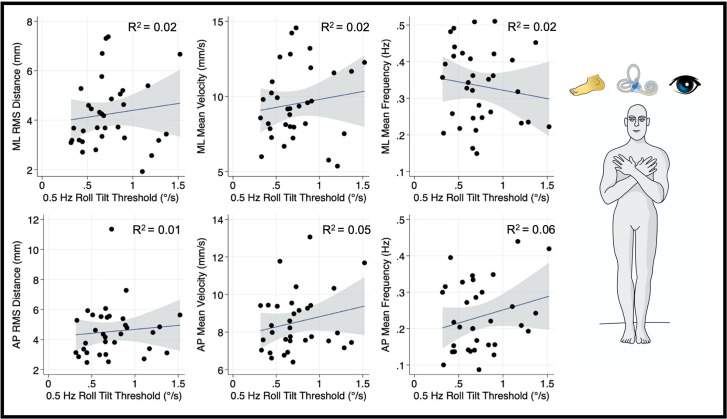
Relationship between 0.5 Hz roll tilt thresholds and RMSD, mean velocity, and mean frequency for both the mediolateral plane (top) and anteroposterior plane (bottom) during an “eyes open, firm” balance task. To the right of the plots, the task and relevant balance control systems are displayed, showing the presence of proprioceptive and visual, as well as vestibular, cues in the “eyes open, firm” condition. Each data point represents one individual’s performance. The blue line shows a linear fit with the 95% CI depicted by the gray shaded region. Unlike the “eyes closed, foam” condition, no significant linear effects were observed between RMSD, MV, or MF and 0.5 Hz roll tilt thresholds. MF, mean frequency; MV, mean velocity; RMSD, root mean square distance.

Our primary univariate analyses that assessed the relationship between vestibular noise, which we posit results from canal-otolith integration (i.e., 0.5 Hz roll tilt thresholds) and mediolateral postural sway were repeated for a balance task that relies minimally on vestibular cues (i.e., eyes open, firm surface; Figure 6). This focused analysis allowed us to assess if the statically significant relationship between vestibular perceptual thresholds at 0.5 Hz and postural sway is constrained to conditions where vestibular cues are prioritized for postural control. Unlike the “eyes closed, on foam” condition, for the CoP metrics calculated from the “eyes open, firm surface” condition, 0.5 Hz roll stilt thresholds did not demonstrate a statistically significant correlation with the mediolateral RMSD (*β* = 0.028, *p* = 0.49, 95% CI: −0.05–0.11), MV (*β* = 0.017, *p* = 0.45, 95% CI: −0.029–0.064), or MF (*β* = 0.41, *p* = 0.45, 95% CI: −1.5–0.68). This is consistent with the hypothesis that 0.5 Hz roll tilt thresholds and postural sway, when assessed in the presence of degraded visual and proprioceptive information, are each influenced by a shared noise source.

**Figure 7:**
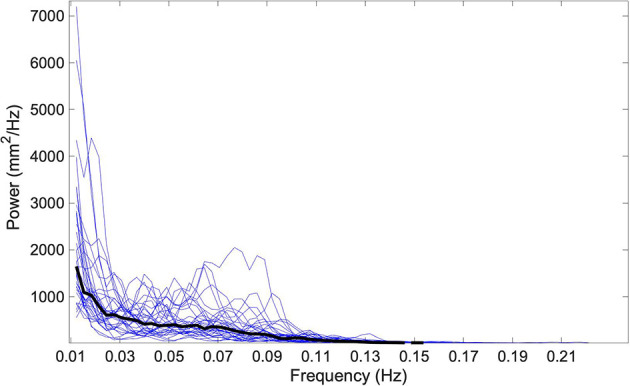
The one-sided power spectral density (PSD) of the ML CoP tracing is shown using blue traces for each of the 33 subjects. The median spectral density calculated at each discrete frequency is shown by a solid black line. 95% of the power in the CoP signal is housed below 0.11 Hz; this is below the frequency for any of the roll tilt perceptual thresholds that were measured (0.2, 0.5, and 1 Hz). CoP, Center of Pressure; ML, mediolateral.

Finally, to determine if the relationship between 0.5 Hz roll tilt thresholds and postural sway was instead the result of a shared dominant frequency (i.e., 0.5 Hz), we performed a spectral analysis of the mediolateral and anteroposterior “eye closed, on foam” CoP data. Power spectral density (PSD) of the ML and AP CoP traces revealed that 95% of the power in the CoP signal resided below 0.11 ± 0.011 and 0.12 ± 0.018 Hz respectively (Figure 7). This supports the supposition that the correlative relationship between the perception of 0.5 Hz tilt stimuli and postural sway was not reflective of a shared dominant frequency, but instead supports that both share a common underlying physiologic element, herein hypothesized to be noise resulting from the temporal integration of noisy canal and otolith cues.

## Discussion

Our data show that vestibular noise demonstrates a significant, positive association with postural sway variability. Specifically, increases in 0.5 Hz roll tilt thresholds, which quantify vestibular perceptual noise, were accompanied by increases in sway. As this positive correlation with roll tilt thresholds was observed only at 0.5 Hz, it suggests that the association between postural sway and vestibular noise may be due to the shared influence of noise resulting from the temporal integration of noisy canal and otolith signals. In addition, this relationship appears to be greatest when: (1) the sway plane is concordant with the direction of the roll tilt threshold stimulus, implying an influence of roll tilt vestibular noise on the spatial control of posture, and (2) quiet stance balance conditions where vestibular cues are prioritized and visual and proprioceptive cues are degraded (i.e., standing on foam with eyes closed). Finally, it appears that variability in the displacement of the body in space (i.e., RMSD) is most affected by vestibular noise, as statistically significant relationships were not apparent when the mean velocity (MV) or the mean frequency (MF) of the CoP were regressed on roll tilt thresholds.

### Temporal Integration and Spatial Control of Posture

An underappreciated role of the vestibular system is its capacity to provide a gravity-referenced estimate of one’s position in space during complex, dynamic tasks (Merfeld, 1995; Glasauer and Merfeld, 1997; Angelaki and Cullen, 2008). The dynamic nature of vestibular function implies a temporal requirement whereby the vestibular system must sense the motion, integrate multiple self-motion cues, and continuously generate an appropriate behavioral output. As a result, the direction recognition task described herein inherently requires the vestibular system to possess the capacity to account for changes in stimulation over time in order to generate a precise estimate of the self-motion cue. Our results show that during a dynamic 0.5 Hz roll tilt motion, lasting two seconds, the precision by which the vestibular system integrates velocity cues from the canals with gravitational cues from the otoliths contributes significantly to one’s ability to control their body in space during an “eyes closed, on foam” balance task where vestibular cues dominate. The implications for this finding are notable, as it suggests that the imprecision in these complex, time-dependent computations may contribute to the control of balance and may have implications for alternative sequalae of vestibular impairment such as gaze instability, cognitive impairment, and autonomic dysregulation. Further, in our multivariable regression models where we controlled for elements common to the three threshold frequencies (e.g., cognition, tactile inputs from the motion device), we still found a significant relationship between 0.5 Hz thresholds and mediolateral postural sway, suggesting that noise associated with the temporal integration of noisy canal and otolithic cues may represent a critical element contributing to variability in postural sway when visual and kinesthetic cues are unreliable, degraded, or unavailable.

### Interpretation of the Frequency Effect

Lim et al. (2017) showed that on average, the dynamic angular velocity cue from the canals, and the tilt cue from the otoliths were optimally integrated during roll tilt at frequencies between 0.2 Hz and 0.5 Hz using an optimal Kalman filter model. Our finding that 0.5 Hz thresholds correlated with postural sway suggests that noise following the temporal integration of noisy canal and otolithic cues may affect postural control. The absence of a significant correlation between postural sway and 0.2 Hz thresholds, where canal-otolith integration presumably still occurs, suggests that the relative reliability of otolith and canal cues may influence this relationship.

At 0.2 Hz, the amplitude of tilt for a given velocity threshold is increased relative to 0.5 Hz (Figure 1), and as a result, 0.2 Hz roll tilt leads to greater stimulation of the tilt-sensitive otolith organs. The increased use of the otolith-derived tilt cue at 0.2 Hz is likely also accentuated by the decreased perceptual sensitivity to the canal-derived rotation cues at frequencies below 0.44 Hz (Lim et al., 2017). Therefore, we posit that the selective correlation between postural sway and roll tilt thresholds at 0.5 Hz, suggests that postural control is preferentially influenced by noise resulting from the temporal integration of noisy otolith and canal signals, rather than the noise in the otolith signal alone. The absence of a correlation between postural sway and 1 Hz roll tilt thresholds supports the supposition that the association between 0.5 Hz thresholds and postural sway reflects the influence of noise resulting from the integration of canal and otolith signals, rather than the central processing of the canal signal. Further, our data showing that more than 95% of the power in the postural sway signal was below 0.5 Hz (Figure 7), suggests that the selective relationship of 0.5 Hz thresholds was not simply reflective of a shared dominant frequency between the two tasks.

### Comparison to Past Empirical Studies

This effort represents one of only three datasets to compare balance to measures of vestibular perceptual noise, as quantified by vestibular thresholds. Karmali et al. (2017) analyzed data collected by Bermúdez Rey et al. (2016) and showed that 0.2 Hz roll tilt thresholds were significantly associated with the likelihood of completing the same “eyes closed, on foam” balance task (Bermúdez Rey et al., 2016; Karmali et al., 2017). While we similarly found a correlation between roll tilt thresholds and balance performance, we did not identify a significant effect for 0.2 Hz thresholds and only saw a relationship at 0.5 Hz. This may reflect differences in the study populations as we only enrolled young healthy adults, while the previous dataset assessed a wide age range (18–89 years) to capture the effects of healthy aging. Another difference, which may also reflect this difference in age of the populations, is that all 33 subjects in our study could complete the “eyes closed, on foam” balance task for 60 s, whereas Bermúdez Rey et al. (2016) reported that only 70/99 could stand in this same condition for 30 s (Bermúdez Rey et al., 2016). The difference in findings between our results and those of Karmali et al. (2017) may also have been due to the methods used to quantify balance performance. While we assessed continuous measures of the CoP quantified using a force plate, Bermúdez Rey et al. (2016) did not utilize a force plate, instead using a categorical “pass/fail” measure (Bermúdez Rey et al., 2016). Thus, 0.2 Hz thresholds may correlate specifically with age-related imbalance or may be reserved for more severe balance impairment, as represented by the inability to complete the aforementioned balance task.

In a more recent publication, Karmali et al. (2021) compared an expanded battery of vestibular thresholds to static postural sway, as well as computerized dynamic posturography, in a sample of healthy adults (21–61 years old; Karmali et al., 2021). They found that only interaural translation thresholds (performed at 1 Hz) were significantly correlated with postural sway (Karmali et al., 2021). A relationship between roll tilt thresholds and postural control was not identified; however, roll tilt thresholds were only quantified at 0.2 and 1 Hz, and not 0.5 Hz, and thus their findings are consistent with the findings reported here. Additionally, analogous to the selective correlation shown here between 0.5 Hz roll tilt and sway during the “eyes closed, on foam” condition, Karmali et al. (2021) similarly showed that correlations between thresholds and sway were strengthened in the conditions where proprioceptive cues were degraded.

The common directionality between interaural (left/right) translation thresholds and dynamic roll tilt, with both occurring in the mediolateral direction, is also worthy of consideration. While we showed that the effect of 0.5 Hz roll tilt was specific to postural sway in the mediolateral direction, Karmali et al. (2021) instead showed that interaural translation thresholds also correlated with postural sway in the anteroposterior direction. Thus, noise associated with the processing of otolith-derived linear acceleration signals, as reflected by interaural translation thresholds, may more generally influence the postural control system, whereas noisy canal-otolith integration may be specific to the spatial control of the body in the corresponding plane of motion. We do note that while the association between 0.5 Hz roll tilt thresholds and AP RMSD in our study was not significant, we did see a positive linear association which may have reached significance with a larger sample size, and thus we cannot rule out that roll tilt thresholds may also more generally predict postural sway in alternative planes.

We also highlight that the findings of Karmali et al. (2021) are not incongruent with the proposed mechanism linking canal-otolith integration to impaired postural control. During interaural translations, the otolith organs encode the net change in gravitoinertial force but cannot discern if the acceleration cues resulted from the effects of gravity, such as during a tilt of the head to the right, or due to a linear acceleration of the head to the left. Thus, the semicircular canals, yielding a signal that indicates an absence of rotation about an earth horizontal axis (i.e., no tilt), are required, analogous to during dynamic roll tilt, to dynamically update internal models within the central nervous system to permit the appropriate perception of the translation stimulus.

### Comparison to Theoretic Noise Parameters

Maurer and Peterka (2005) used simulations of CoP data to compare the traditional postural sway metrics reported here (i.e., RMSD, MV, and MF) to model parameters derived from a closed loop model of postural control. They found significant correlations between the noise parameter from their model (i.e., a Gaussian signal disturbing the balance system) and the RMSD and MV, but not MF, of the CoP (Maurer and Peterka, 2005). Here, we show that empirical measures of vestibular noise similarly display a significant association with the RMSD and no significant association with the MF of the CoP; however, unlike the theoretical model, none of our vestibular threshold metrics were significantly correlated with the MV, despite a positive association (*p* = 0.079) between 0.5 Hz thresholds and mediolateral MV. As these simulations by Maurer and Peterka were based on the performance of older adults, the effects of vestibular noise on CoP velocity may be emphasized by aging, which might have been tempered in our analysis of young healthy adults. While speculative, we posit that the correlations of both the empirical data (reported herein) and model-based noise parameters (Maurer and Peterka, 2005) with similar CoP metrics (RMSD, MV) suggest that postural control and roll tilt thresholds are influenced by a shared source of vestibular noise and that the selective correlation to 0.5 Hz roll tilt thresholds suggests that the common element is noise resulting from the temporal integration of noisy canal and otolith cues required to best estimate tilt.

### Limitations

Here we define self-motion perceptual thresholds as measures of vestibular sensory noise, however, we acknowledge the presence of extra-vestibular inputs during these tasks. The notion that direction recognition thresholds rely predominantly upon vestibular cues is supported by past data showing that perceptual thresholds were 2.5–56.8 times higher in patients with absent bilateral vestibular function (due to bilateral labyrinthectomy/neurectomy; Valko et al., 2012). Due to time constraints (i.e., limiting the battery to <2 h) we only captured thresholds across a narrow range, and thus future studies would benefit from utilizing both higher (>1 Hz) and lower (<0.2 Hz) frequencies to better isolate canal and otolith noise relative to the centrally integrated canal-otolith signal. As well, future studies could further explore the directional specificity of the relationship between canal-otolith integration and postural sway by quantifying vestibular thresholds in additional planes of motion (e.g., pitch tilt). As a final limitation, to avoid “fishing”, we pre-selected only three of the many (>15) possible metrics commonly used to describe the CoP (Prieto et al., 1996). Yet, we highlight that previous data (Maurer and Peterka, 2005) has shown that CoP metrics naturally separate into three distinct groups of highly correlated variables. To avoid redundant analyses and associated “fishing” for statistically significant findings, we a priori chose to only use one CoP measure from each category [i.e., displacement (RMSD), velocity (MV), and frequency (MF)] in this study.

## Conclusion

Our data showed that vestibular noise resulting from the temporal integration of noisy canal and otolith signals is significantly and positively associated with the amount of variability in postural sway in the corresponding mediolateral plane. These findings suggest that the precision by which the vestibular system integrates canal and otolith signals over time significantly impacts the ability to control the position of the body in space.

## Data Availability Statement

The raw data supporting the conclusions of this article will be made available by the authors, without undue reservation.

## Ethics Statement

The studies involving human participants were reviewed and approved by The Ohio State University Institutional Review Board. The patients/participants provided their written informed consent to participate in this study. Written informed consent was obtained from the individual(s) for the publication of any potentially identifiable images or data included in this article.

## Author Contributions

AW, MK, and DM conceptualized the experiment. AW and MK collected the data. AW analyzed the data and wrote the initial draft of the manuscript. MK and DM made edits and substantive contributions to the manuscript. All authors contributed to the article and approved the submitted version.

## Conflict of Interest

The authors declare that the research was conducted in the absence of any commercial or financial relationships that could be construed as a potential conflict of interest.

## Publisher’s Note

All claims expressed in this article are solely those of the authors and do not necessarily represent those of their affiliated organizations, or those of the publisher, the editors and the reviewers. Any product that may be evaluated in this article, or claim that may be made by its manufacturer, is not guaranteed or endorsed by the publisher.
